# Assessment of maxillofacial trauma in emergency department

**DOI:** 10.1186/1749-7922-9-13

**Published:** 2014-01-31

**Authors:** Engin D Arslan, Alper G Solakoglu, Erdal Komut, Cemil Kavalci, Fevzi Yilmaz, Evvah Karakilic, Tamer Durdu, Muge Sonmez

**Affiliations:** 1 Emergency Department, Ankara Numune Training and Research Hospital, Altındağ, 06100 Ankara, Turkey; 2 Emergency Department, Başkent Univercity Hospital, Çankaya, 06350 Ankara, Turkey; 3 Radiology Department, Ankara Numune Training and Research Hospital, Altındağ, 06100 Ankara, Turkey

**Keywords:** Maxillofacial trauma, Mid face fracture, Emergency department

## Abstract

**Introduction:**

The incidence and epidemiological causes of maxillofacial (MF) trauma varies widely. The objective of this study is to point out maxillofacial trauma patients’ epidemiological properties and trauma patterns with simultaneous injuries in different areas of the body that may help emergency physicians to deliver more accurate diagnosis and decisions.

**Methods:**

In this study we analyze etiology and pattern of MF trauma and coexisting injuries if any, in patients whose maxillofacial CT scans was obtained in a three year period, retrospectively.

**Results:**

754 patients included in the study consisting of 73.7% male and 26.3% female, and the male-to-female ratio was 2.8:1. Mean age was 40.3 ± 17.2 years with a range of 18 to 97. 57.4% of the patients were between the ages of 18–39 years and predominantly male. Above 60 years of age, referrals were mostly woman. The most common cause of injuries were violence, accounting for 39.7% of the sample, followed by falls 27.9% and road traffic accidents 27.2%. The primary cause of injuries were violence between ages 20 and 49 and falls after 50. Bone fractures found in 56,0% of individuals. Of the total of 701 fractured bones in 422 patients the most frequent was maxillary bone 28,0% followed by nasal bone 25,3%, zygoma 20,2%, mandible 8,4%, frontal bone 8,1% and nasoethmoidoorbital bone 3,1%. Fractures to maxillary bone were uppermost in each age group.

8, 9% of the patients had brain injury and only frontal fractures is significantly associated to TBI (p < 0.05) if coexisting facial bone fracture occurred. Male gender has statistically stronger association for suffering TBI than female (p < 0, 05). Most common cause of TBI in MF trauma patients was violence (47, 8%).

158 of the 754 patients had consumed alcohol before trauma. No statistically significant data were revealed between alcohol consumption gender and presence of fracture. Violence is statistically significant (p < 0.05) in these patients.

**Conclusion:**

Studies subjected maxillofacial traumas yield various etiologic factors, demographic properties and fracture patterns probably due to social, cultural and governmental differences. Young males subjected to maxillofacial trauma more commonly as a result of interpersonal violence.

## Introduction

The incidence and epidemiological causes of maxillofacial (MF) trauma and facial fractures varies widely in different regions of the world due to social, economical, cultural consequences, awareness of traffic regulations and alcohol consumption. Reports from distinct regions in Turkey also have different etiological findings [1,2]. According to the studies in developed countries assault is the leading cause of facial fractures followed mostly by motor vehicle accidents, pedestrian collisions, stumbling, sports and industrial accidents but the leading cause shifts to road traffic accidents in underdeveloped or developing areas of the world followed by assaults and other reasons including warfare [3-9].

Diagnosis and management facial injuries are a challenge particularly in the setting of coexisting polytrauma in emergency department. Our goal is to broaden clinical data of MF trauma patients for public health measures. It is our credence that broader knowledge of MF trauma patients’ epidemiological properties and trauma patterns with simultaneous injuries in different areas of the body may help emergency physicians to deliver more accurate diagnosis and decisions. In this study we analyze etiology and pattern of MF trauma and coexisting injuries if any.

## Patients and methods

In the study MF injuries were diagnosed after evaluation of the patients’ history, physical examination, forensic record and radiological studies. Patients with isolated nasal and dentoalveolar fracture were excluded and in patients with suspected more severe facial injuries, maxillofacial CT scans were performed as proposed by our hospitals clinical policy. We retrospectively evaluated patients referred to our emergency department (ED) between 2010 March and 2013 March whose maxillofacial CT scans were obtained. Our study’s variables are presented as; age, gender, cause of injury, site of injury, alcohol consumption, coexisting intracranial, cervical, orthopedic, abdominal injuries and mortality if any. During the analyses Mid-face region injuries were classified as Le Fort I, Le Fort II, Le Fort III, blow out, zygomaticomaxillary complex, nasorbitoethmoid complex and zygomatic arc fractures. Pan-facial fracture is defined as fractures affecting all three parts of face (Frontal, mid-face and mandible at the same time). If the patient suffered from multiple fractures, each fracture was analyzed separately and if the patient had traumatic brain injury Glasgow Coma Scale (GCS) was evaluated and GCS was grouped as mild (14–15), moderate (8–13) and severe (3–8). All data was documented on SPSS v.17 and analyzed. Comparisons were made with chi-square test with%95 confidence interval and p values <0, 05 were considered as statistically significant. All authors obey the rules of Helsinki Declaration and no ethic problem exist in the manuscript.

## Results

### Demographic pattern of the patients and trauma mechanisms

556 (73.7%) male and 198(26.3%) female patients were included in the study and the male-to-female ratio was 2.8:1. Mean age was 40.3 ± 17.2 years with a range of 18 to 97 years also mean age of patients with MF fractures were almost the same (40, 06 ± 17, 2). Majority of the patients (n = 432, 57.4%) were between the ages of 18–39 years and predominantly male. Above 60 years of age, referrals were mostly woman.

The most common cause of injuries were violence, accounting for 39.7% (n = 299) of the sample, followed by falls 27.9% (n = 210) and road traffic accidents 27.2% (n = 205). In patients between 20 to 49 years violence was the main cause of injuries, whereas after 50 years old falls were the primary cause of injuries. These associations were found to be statistically significant (p < 0, 0001).

When road traffic accidents were subdivided, motor vehicle accidents have the ratio of 17.7% (n = 134) of all patients, followed by vehicle-pedestrian collisions 8.1% (n = 61) and motorcycle accidents (n = 9) 1.2%. No statistically relevant data were identified between gender, age group and trauma causes. Table 1 illustrates age, gender and trauma mechanism relationships.

**Table 1:** Trauma mechanisms according to age and gender

**Ages**	**Gender**	**Violence**	**Stumble and fall**	**Road traffic accidents**	**Strike by object**	**Occupational**	**Explosion**	**Total (%)**
19–30	Male	99	32	59	13	0	1	204 (27.1)
	Female	16	9	17	1	0	0	43 (5.7)
31–40	Male	85	22	30	6	8	2	153 (20.3)
	Female	9	9	13	0	0	1	32 (4.2)
41–50	Male	52	23	19	1	1	0	96 (12.7)
	Female	5	8	13	2	0	0	28 (3.7)
51–60	Male	16	27	14	2	0	0	59 (7.8)
	Female	6	10	17	1	0	0	34 (4.9)
61–70	Male	8	8	5	1	0	0	22 (2.9)
	Female	0	11	4	0	0	0	15 (2.0)
70+	Male	2	13	7	0	0	0	22 (2.9)
	Female	1	38	7	0	0	0	46 (6.1)
Total (%)		299 (39.7)	210 (27.9)	205 (27.2)	27 (3.6)	9 (1.2)	4 (0.5)	754

### MF injury and fracture analyses

#### Fracture, injury patterns, age and cause of injury classification

**Figure 1:**
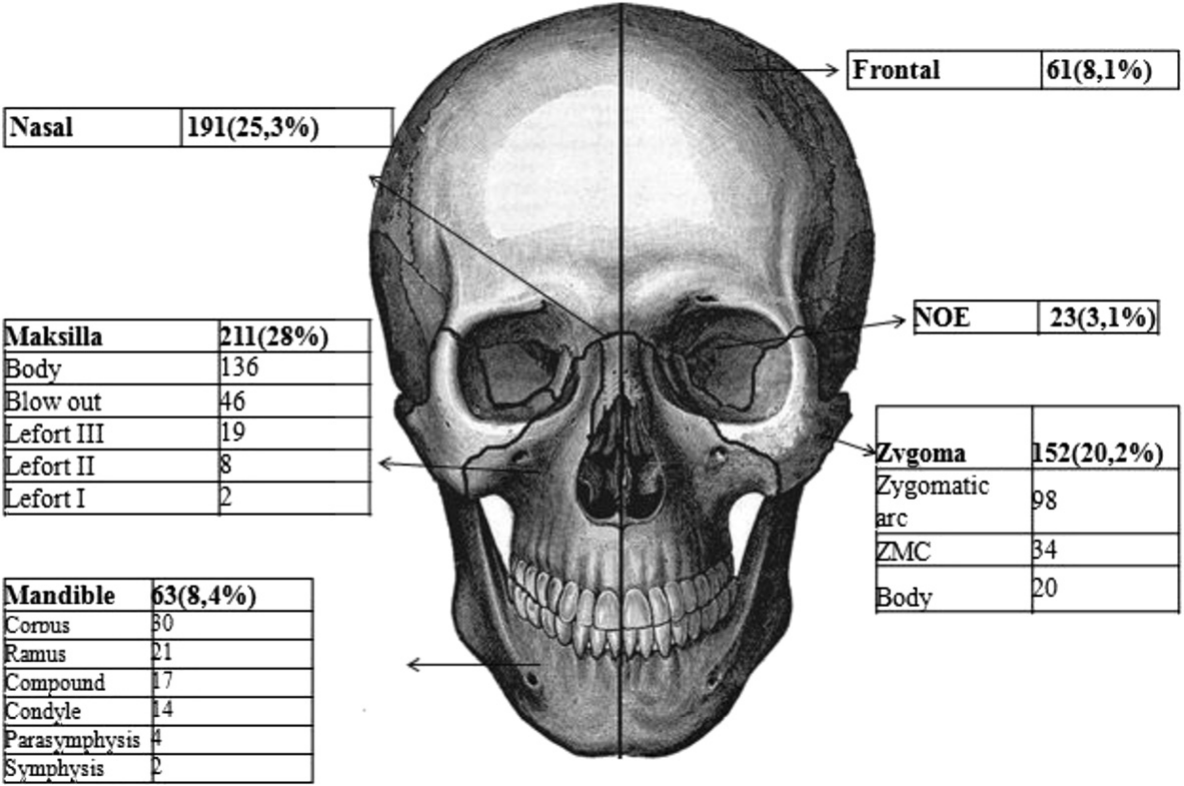
Facial fractures according to anatomical sites.

**Figure 2:**
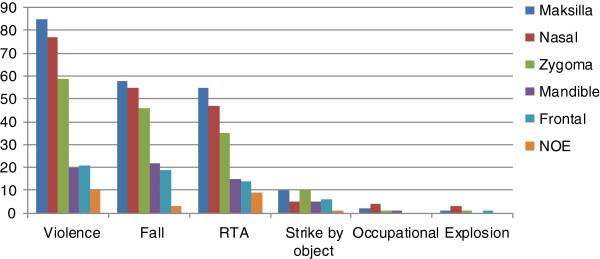
Number of fractured bones according to trauma mechanisms.

Soft-tissue injuries accounted for 44,0% (n = 332), while bone fractures 56,0% (n = 422). Of the total of 701 fractured bones in 422 patients the most frequent was maxillary bone n = 211(28,0%) followed by nasal bone n = 191 (25,3%), zygoma n = 152 (20,2%), the mandible n = 63 (%8,4) frontal bone n = 61 (8,1%) and nasoethmoidoorbital bone n = 23(%3,1). Fractures to maxillary bone were uppermost in each age group. Figure 1 illustrates facial fractures according to anatomical sites and Figure 2 explains the relationship of fractures with trauma mechanisms.

Violence was mostly the cause of nasal, maxillary, zygoma and frontal bone fractures whereas for mandibular fractures main cause was falls. Statistically important trauma mechanism causing any facial bone fractures was not displayed.

#### Fracture analyses according to anatomical sites

##### Mid-facial fractures

In this study there were 385 patients with fractures of the mid-face. Most frequent mid-face fractures were maxillary fractures (27,4%) followed by nasal bone (25,8%) and zygoma (20,2%) fractures. Simultaneous fractures of mid-face including multiple zygoma, maxillary, nasal fractures are classified as combined fractures and constitute 11,7% of patients. For combined fractures most common cause is falls. Isolated zygomatic arch fractures were often as a result of violence and falls and related in 19-30 age group with (p <0, 0001). Table 2 details the relationship with trauma mechanism and fracture sites with special considerations. Multiple facial bone fractures in same patients must be considered.

**Table 2:** Special midfacial fractures according to trauma mechanism

	**RTA**	**Violence**	**Occupational**	**Falls**	**Explosion**	**Struck by object**	**Total**
Lefort I	0	1	0	0	0	0	1
Lefort II	6	1	0	1	0	0	8
Lefort III	9	5	0	5	0	0	19
Blowout	14	15	3	10	1	3	46
ZMC	10	7	0	16	0	1	34
Zygomatic arc	25	34	1	35	0	3	98
NOE	8	8	1	6	0	0	23

##### Mandibular fractures

A total of 63 patients with mandibular fractures were documented. The main fracture site was mandibular corpus (28,5%) followed by ramus (23,8%). Ratio of patients suffering from fractures affecting more than one anatomical mandibular sites is 26,9%. Most common combined fracture of mandible was ramus and angle fracture, effecting 17, 4% of patients. The fractures were generally caused by falls (34.5%), followed by violence (31.1%).

### Fractures and coexisting traumas

#### MF traumas coexisting with traumatic brain injury and skull fractures

Of all the patients 8, 9% had brain injury whereas RTA patients had ratio of 13, 7%. Only frontal fractures are significantly associated to Traumatic Brain Injury (TBI) (p < 0.05) if coexisting facial bone fracture occurred and Cramer’s V and Phi value is above 0.3. Male gender has statistically stronger association for suffering TBI than female (p < 0, 05). Most common cause of TBI in MF trauma patients was violence (47, 8%) followed by falls (28, 4%) and road traffic accidents (RTA) (20, 9%). Most common TBI was subarachnoid hemorrhage (44,8%), followed by contusions (22,4%), epidural hematoma (20,9%), pnemocephalus (19,4%), subdural hematoma (16,4% ) and diffuse axonal injury (6%). Of the 68 patients with TBI 17 patients had suffered from severe brain traumatic brain injury and 6 of them died of TBI. 33 patients had mild and 18 had moderate brain trauma and admitted to brain surgery ward for observation and surgery if necessary. Multiple TBI patterns in same patients must be considered.

#### Traumas to non-facial areas and hospital mortality

172 (22,8%) patients suffered from 232 total injuries both to cranium and body. Additional body trauma rather than cranium occurred in 15, 4% (n = 116) of patients. Of these; injuries to upper extremity, lower extremity, chest, pelvis and abdomen were seen in 5,8% (n = 44), 4,6% (n = 35), 4% (n = 30), 1, 9% (n = 17) and 1, 6% (n = 12) of patients respectively.

In RTA victims the ratios vary, total of 30,7% (n = 63) patients suffered from coexisting trauma and injury of the upper extremity was noticed in 12, 2% (n = 25), followed by injury to lower extremity in 11, 7% (n = 24) chest in 10, 7% (n = 22) pelvis in 4, 9% (n = 10), abdomen in 3, 9% (n = 8). Table 3 illustrates details of injury patterns with co-existing trauma.

**Table 3:** Fractures and injury patterns in patients with coexisting maxillofacial trauma

		**n of patients**	**% of patients**
Orthopaedic injuries	Hand/wrist	17	9,8
	Forearm	16	9,3
	Femur	16	9,3
	Tibia/Fibula	16	9,3
	Humerus	11	6,3
	Clavicle/Scapula	10	5,8
	Foot/Ankle	9	5,2
	Lumber vertebra	3	1,7
Abdominal/Pelvic	Pelvis fracture	13	7,5
	Spleen hematoma	5	2,9
	Liver hematoma	4	2,3
	Pelvis hematoma	2	1,1
	Gastric perforation	2	1,1
	Retroperitoneal hematoma	1	0,5
Torso injuries	Clavicle/Scapula fracture	10	5,8
	Pnemothorax/Hemothorax	11	6,3
	Costa fracture	7	4,0
	Pulmonary contusion	2	1,1
		**n**	**% of patients with TBI**
TBI’s	Subarachnoid haemorrhage	30	44.1
	Brain contusion	15	22
	Epidural haemorrhage	14	20.5
	Pnemocephalus	13	19.1
	Subdural haemorrhage	11	16.1
	Diffuse axonal injury	4	5.8

A total of 24 patients were intubated during the study period. 17 patients were intubated because of severe traumatic brain injury and 7 from trauma complications such as pnemothoraces, hemorrhagic shock etc. Of the 17 severe TBI patients only 2 of them had isolated sagittal maxillary fracture and 1 had soft tissue injury. 3 of the patients had panfacial trauma with Lefort III type maxillary fracture where as 11 patients had compound midfacial and/or mandibular fracture.

6 of the admitted patients died from TBI, 1 from ICU complication and 2 from internal bleeding.

### Injury and association with alcohol consumption

158 of the 754 patients had consumed alcohol before trauma. No statistically significant data were revealed between alcohol consumption gender and presence of fracture. Trauma mechanism of facial injury in intoxicated patients was distributed almost evenly, most common cause is violence and compared to other causes, suffering from violence is statistically higher (p < 0.05) furthermore young male group (age between 19-30) is consuming more alcohol compared to other age groups in same gender (p < 0.001).

## Discussion

Trauma is the leading cause of deaths occurred in first 40 years of life and it is well known that MF injuries are frequently seen in polytrauma victims. MF region includes organs executing essential functions of the body like respiration, speech, mastication, vision, smelling so special attention must be paid in case of facial trauma. Advanced trauma life support (ATLS) principles must be applied for the initial assessment of all MF injury victims as in any trauma patient. The most important sequence of ATLS is maintenance of airway patency in these patients. Airway compromise should occur due to tongue falling back, hemorrhage to oropharyngeal region, foreign bodies, mid facial fractures themselves. If possible endotracheal intubation is the preferred method to establish airway patency as no chance to intubate, crichothyroidotomy can be performed particularly in comatose patients [10].

In this study we assessed the epidemiology of MF injuries in emergency department as first contact of injured patients and analyzed 754 patients with facial injuries caused by various mechanisms. According to the Turkish Statistical Institute’s data in 2013, Ankara has a population of 4.965.552 and is the second largest city in Turkey. Our Research and Training hospital is one of the historical hospitals in Ankara with a level-1 trauma center and gets referrals from Ankara and other neighboring cities. Our population and trauma mechanisms are distinct from other studies executed in Middle East countries. There were 556 (%73.7) male and 198 (%26.3) female and the male-to-female ratio was 2.8:1 and assaults are seen as primary cause of trauma mechanism. In our neighboring Middle East countries male to female ratios varies from 4.5:1 to 11:1 [9,11-13]. Segregation of women from social life in these countries may be the cause of disproportionate gender distribution. Our gender distribution is more likely to urbanized European countries particularly since woman rights are relatively well established in Turkey [5,6].

Most common age group encountering MF trauma is 19–30 age group and that seems to be correlated with the other studies and as exposed by the other studies higher age is more correlated to falls and younger age is more inclined to assaults and road traffic accidents [5,8]. In our investigation falls are the primary cause of injury in females accounting for 42,9% of the samples whereas assaults lead in males (%47, 1).

Our trauma mechanism analyses are also characteristic for Turkey’s unique sociocultural background. Studies mentioned above from eastern countries reveal that most common trauma mechanism is road traffic accidents. We believe lack of traffic regulations in these countries may be the cause of high ratio of RTA’s. In our study most common trauma mechanisms are assaults followed by falls. But our populations’ assault rate is not as high as our western neighbor Bulgaria [6]. Another study in Ankara, conducted in our hospitals plastic surgery department by Aksoy et all at late 1990’s revealed notable differences with our study that trauma pattern shifted from road traffic accidents to assaults in our hospital [1]. For the past 20 years Turkey is adopting traffic regulation laws including seat belt usage and driver side airbag implantation on cars which is shown by Mouzakes et al to protect patients from MF trauma [14]. Although it seems hard to postulate we estimate that people’s compliance to new laws may be relatively lower than European countries.

Plenty of studies were executed for fracture patterns in MF trauma in oral and facial departments throughout the world [6,7,9,13,15]. These studies including the Aksoy et al reported that mainly mandibular and zygomatic bones were fractured bones [1]. In our study we found that most frequent fractured bone was maxillary bone (28, 0%) followed by the nasal bone (25, 3%). To minimalize the missing mid-facial fractures that cannot be diagnosed by physical examination or conventional direct graphs, we confirmed the fractures by coronal and axial maxillofacial CT scans but we did not perform CT scan in patients whom we consider mild facial trauma. We believe that’s the basis of relatively low ratio of nasal fracture for ER patient sample.

Zygoma fractures are mostly seen in young male patients whose life style are at high risk for trauma and in our study we observed that isolated zygomatic arch fractures were usually because of violence and falls. Also zygomatic arc fractures are associated in young male age group. Another study from Brazil focusing on zygoma fractures demonstrated that falls and assaults were the leading cause of injuries, compatible with our study. Age group and gender distribution is alike with Brazil study [16].

EDs serve as the first point of entry into the hospital system for a significant percentage of patients seeking treatment for MF injuries [17]. Furthermore we suppose that majority of emergency physicians deal with simple maxillary and nasal bone fractures without consultations that may explain the differences in fracture distribution between ED and oral and facial surgery departments. One of the few studies from ED was performed in Tehran explains about facial trauma epidemiology [18]. Contrary to our results they have found that mandibular and nasal bones fractures were most common. We believe this difference is due to their patient universe which includes more severe trauma patients who requires 24 hour observation period.

A few study tried to correlate TBI with facial lesions to open a pathway to emergency physicians’ clinical decisions. In our study there was no association between, trauma mechanism and gender to TBI. Frontal fractures with coexisting fractures in mid face and mandible caries higher risk for TBI so should be managed cautiously.

There is also a lack of studies involving MF trauma to non-facial areas of body and mortality, in our study we have found total of 15.3% of patients suffered coexisting trauma. Study from India [19] points out that mostly head and orthopedic injuries are seen in MF trauma patients. Indian study reports high coexisting trauma rate of 25.6%. We believe that this ratio is due to high ratio of road traffic accident victims in that study. In our study road traffic accident patients have ratio of 30, 7% additional trauma with high ratio of orthopedic and head injuries in line with Indian study.

Alcohol use is another reason for MF traumas leading to hostile behavior causing violence and careless driving causing RTA in addition to that intoxicated patients are usually difficult to examine and small fractures in intoxicated patients can easily be misdiagnosed. Reduction of drunk drivers reduces MF trauma severity and the association of alcohol and interpersonal violence is well recognized [20,21]. We have found that 158 of the 754 patients were intoxicated before trauma. This relatively high ratio for a highly Muslim populated country can be explained by our hospitals place which is famous for its night-life like Jeju [3]. Alcohol consumption declines rapidly in our eastern neighbors [22].

## Conclusion

MF trauma management is sometimes challenging in emergency room. Knowing the MF trauma presentations, concomitant non facial injuries and TBI patterns are important for emergent management. To our knowledge common literature lacks studies from ED. We believe for MF trauma epidemiology, ED study results are more reliable in the light of information above. Further studies are needed to improve our hypothesis.

## Abbreviations

ED: Emergency department; MF: Maxillofacial; RTA: Road traffic accidents; TBI: Traumatic brain injury.

## Competing interests

The authors declare that they have no competing interests.

## Authors’ contributions

EDA and AS conceived of the study, participated in the design of the study and drafted the manuscript. CK and EK participated in the sequence alignment and performed the statistical analysis EK carried out the imagining studies, and helped to draft the manuscript. FY, TD, MS participated in its design and coordination. All authors read and approved the final manuscript.
